# A LDA-based approach to promoting ranking diversity for genomics information retrieval

**DOI:** 10.1186/1471-2164-13-S3-S2

**Published:** 2012-06-11

**Authors:** Yan Chen, Xiaoshi Yin, Zhoujun Li, Xiaohua Hu, Jimmy Xiangji Huang

**Affiliations:** 1 State Key Laboratory of Software Development Environment, Beihang University, Beijing 100191, China; 2 Beijing Key Laboratory of Network Technology, Beihang University, Beijing, China; 3 College of Information Science and Technology, Drexel University, Philadelphia, PA, USA; 4 School of Information Technology, York University, Canada

## Abstract

**Background:**

In the biomedical domain, there are immense data and tremendous increase of genomics and biomedical relevant publications. The wealth of information has led to an increasing amount of interest in and need for applying information retrieval techniques to access the scientific literature in genomics and related biomedical disciplines. In many cases, the desired information of a query asked by biologists is a list of a certain type of entities covering different aspects that are related to the question, such as cells, genes, diseases, proteins, mutations, etc. Hence, it is important of a biomedical IR system to be able to provide relevant and diverse answers to fulfill biologists' information needs. However traditional IR model only concerns with the relevance between retrieved documents and user query, but does not take redundancy between retrieved documents into account. This will lead to high redundancy and low diversity in the retrieval ranked lists.

**Results:**

In this paper, we propose an approach which employs a topic generative model called Latent Dirichlet Allocation (LDA) to promoting ranking diversity for biomedical information retrieval. Different from other approaches or models which consider aspects on word level, our approach assumes that aspects should be identified by the topics of retrieved documents. We present LDA model to discover topic distribution of retrieval passages and word distribution of each topic dimension, and then re-rank retrieval results with topic distribution similarity between passages based on *N*-size slide window. We perform our approach on TREC 2007 Genomics collection and two distinctive IR baseline runs, which can achieve 8% improvement over the highest Aspect MAP reported in TREC 2007 Genomics track.

**Conclusions:**

The proposed method is the first study of adopting topic model to genomics information retrieval, and demonstrates its effectiveness in promoting ranking diversity as well as in improving relevance of ranked lists of genomics search. Moreover, we proposes a distance measure to quantify how much a passage can increase topical diversity by considering both topical importance and topical coefficient by LDA, and the distance measure is a modified Euclidean distance.

## Background

Traditional information retrieval (IR) system should respond with a ranked list of retrieved documents or passages to users, according to their probabilities of relevance to the query. The model only concerns with the relevance between retrieved documents and user query, but does not take redundancy between retrieved documents into account. The retrieved documents with similar contents thus tend to appear over and over again. Ideally, in order to provide a comprehensive picture of all interpretations to the query, it would be better for an information retrieval system to return a ranked list of retrieved documents or passages taking both relevance and diversity into account.

For genomics information retrieval, the problem is particularly prominent, on account of immense data and tremendous increase of genomics and biomedical relevant publications. The wealth of information has led to an increasing amount of interest in and need for applying information retrieval techniques to access the scientific literature in genomics and related biomedical disciplines. In many cases, the desired information of a question (query) asked by biologists is a list of a certain type of entities covering different aspects that are related to the question [1], such as cells, genes, diseases, proteins, mutations, etc. Hence, it is important of a biomedical IR system to be able to provide relevant and diverse answers to fulfill biologists' information needs. In recent years, the "aspect retrieval" was proposed in TREC Genomics tracks. The aim of the aspect retrieval task is to promote retrieval ranking diversity in the ranked list of retrieved passages. Aspects of a retrieved passages could be a list of named entities or MeSH terms [2], representing answers that cover different portions of a full answer to the query. Aspect Mean Average Precision (MAP) [2] was defined in the Genomics tracks. Its purpose is to study how a biomedical retrieval system can support a user to gather information about different aspects of a query. Biomedical retrieval system should return relevant information at the passage level; meanwhile, judges would comprehensively rate the retrieved passages by relevance as well as aspect diversity. Relevant passages that do not contribute any new aspects will not be used to accumulate Aspect MAP. Therefore, Aspect MAP is a measurement for both relevance and diversity of an IR ranked list.

Our work is inspired by several recent papers that concerned with promoting ranking diversity in IR ranked list. The most representative method is maximum marginal relevance (MMR) proposed by Carbonell *et al.*[3]. The MMR method selects a document that has the highest combination of a similarity score with respect to a query and a dissimilarity score with respect to the documents selected at earlier ranks at each iteration. Zhang *et al.*[4] presented four redundancy measures. They modeled relevance and redundancy separately. Since they focused on redundant document filtering, experiments in their study were only conducted on a set of relevant documents. Zhai *et al.*[5] proposed a sub-topic retrieval framework which models relevance and redundancy within the language modeling framework. In particular, they devised several methods based on the Kullback-Leibler divergence measure and a mixture model. The basic idea of above three methods is to penalize redundancy by lowering an item's rank if it is similar to the items already ranked. However, these methods often treat relevance ranking and diversity ranking separately, and sometimes with heuristic procedures. Rianne Kaptein *et al.*[6] employed a top down sliding window to diversify ranked list of retrieved documents. They kept the highest ranked result as is and chose from the next *n *documents the one that maximizes diversity according to some diversity indicators, such as the number of new terms or new links introduced by the next document. A recent study concerning on the Genomics aspect retrieval was conducted by Huang *et al.*[7]. Their experimental results demonstrated that the hidden property based re-ranking method can achieve promising and stable performance improvements. Yin *et al.*[8] proposed a cost-based re-ranking method to promote ranking diversity. This method concerns with finding the passages that cover more different aspects of a query topic. A side effect of these three re-ranking strategies is that they favor long documents, as the long documents tend to contain more distinct terms. In biomedical retrieval domain, Zhu *et al.*[9] proposed a clustering-based ranking algorithm called GRASSHOPPER to promote ranking diversity. GRASSHOPPER is an alternative to MMR and variants with a principled mathematical model and strong empirical performance on artificial data set. Unfortunately, this re-ranking method would reduce their system's performance and decrease the Aspect MAP of the original results for the genomics aspect retrieval [10].

However, the previous work considers the aspects of user query and retrieved documents mainly on word level. In other words, one word or more co-occurrence words are used to identify a specific aspect. For instance, given two retrieval passages: the first one is related to some disease research, in which kidneys of white rats are used as experimental materials; the second one is relevant to subject of kidney transplantation. Obviously, the aspect of kidney occurs in both passages. Under this situation, re-ranking order of the second passage is likely to be reduced because kidney aspect has occurred in the first passage we have observed. However, the second passage is not redundant in fact. The above problems are due to two following reasons: firstly, one or more co-occurrence words in a passage are used to identify the aspect. However, it is common sense for us that a specific word can express more than one latent topics according to different contexts in a passage. In this above case, the word kidney can be used to express the experimental material as well as the object of organ transplant; secondly, words in a passage are considered as independent to each other. However, some potential relationships between words might exist. As shown in the above instance, the word kidney represents two different topics in the two passages, as it has distinctive contexts and relationships with other words. Therefore, it is insufficient to identify aspect on word level.

In this paper, we aim at addressing both above problems simultaneously and assume that aspects should be identified by the topics of retrieved passages. We thus propose an approach which employs Latent Dirichlet Allocation (LDA) [11], a topic generative model, to promote diversity and reduce redundancy in the ranked list for biomedical information retrieval. Specifically, we discover topic distributions of retrieval passages and word distributions of each topic dimension using LDA model, and then re-rank retrieved passages with topic distribution similarity between passages based on a "*N*-size slide window" strategy. Experiments conducted on TREC 2007 Genomics track collection and two very different IR baseline runs demonstrate the effectiveness of our approach. The evaluation results show that our approach can achieve 8% improvement over the highest Aspect MAP reported in TREC 2007 Genomics track. Although the proposed method is not as good as the ones presented in [8] and [12] in terms of MAP performance, it is still promising because we do not employ other resources such as Wikipedia, which is more efficient in data preprocessing. Moreover, we can present aspect probability distributions for each topic.

## Methods

Aspects from retrieved passages can not simply be identified on word level because a specific word can be used to represent more than one topic in different passage contexts. In this case, we assume that aspects should be identified by latent topics hidden in passages which are considered to be more abstract. In the rest of this paper, we use "topic" and "aspect" interchangeable. Furthermore, words in one passage are not independent, which together construct passage topics. It can be also observed that two passages even with the same words can express different topics. We thus assume that latent topics can be identified by word distribution. In this section, we will expound how we use a particular generative model called LDA to discover the topics covered by retrieved passage collection, and illustrate how these topics can be used to improve ranking diversity.

### Aspect discovery and transformation

#### Aspect discovery using LDA

Discovering aspects covered by each retrieved passage is the first step for re-ranking. Recently, a number of approaches [11,13] to modeling document content are proposed and based on the principle that the probability distribution over words in a document can be expressed as a mixture of topics, where each topic is a probability distribution over words. LDA [11] is such a generative probabilistic model of document collection and has been used in many other applications such as computer vision [14], image modeling [15], social tagging system [16], etc. We thus employ this model for aspect discovery. Its basic idea is that documents are represented as random mixtures over latent topics, where each topic is characterized by a distribution over words.

**Figure 1:**
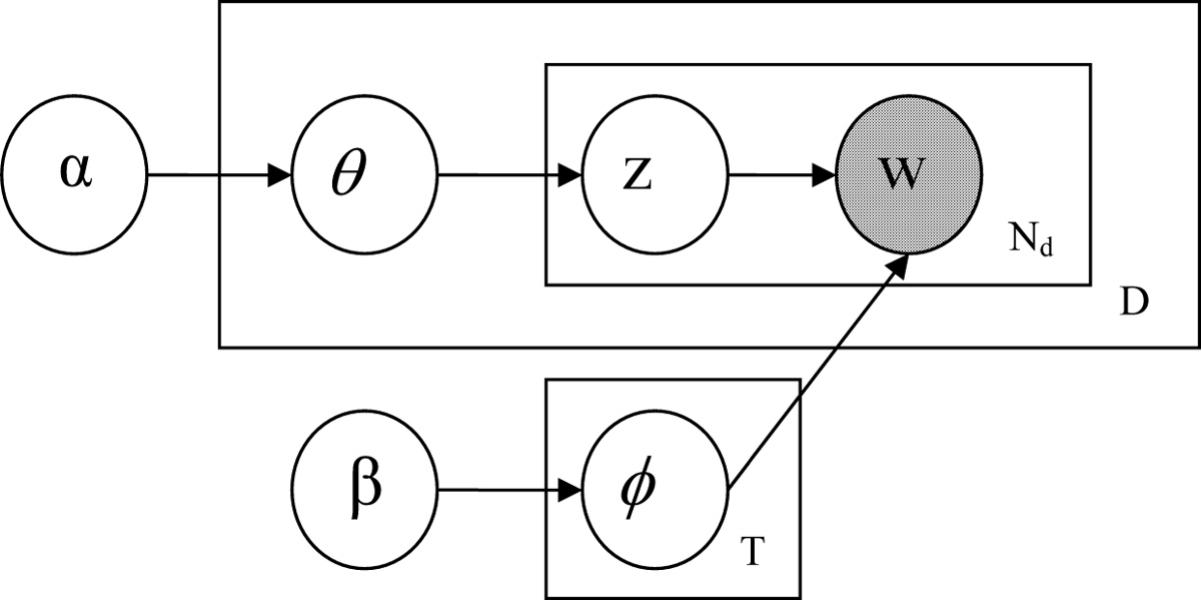
**LDA model**. Figure 1 shows a probabilistic graphical representation of LDA model.

The LDA model is represented (using plate notation) as a probabilistic graphical model in Figure 1. It can be seen clearly from the figure that the LDA representation has three levels and the generation of a document collection is modeled as a three-step process. First, for each document, a distribution over topics is sampled from a Dirichlet distribution. Second, for each word in the document, a single topic is chosen according to this distribution. Finally, each word is sampled from a multinomial distribution over words specific to the sampled topic. In this model, *ϕ *denotes the matrix of topic distributions, with a multinomial distribution over *N *word items for each of *T *topics being drawn independently from a symmetric Dirichlet(*β*) prior. *θ *is the matrix of document-specific mixture weights for these *T *topics, each being drawn independently from a symmetric Dirichlet(*β*) prior. For each word, *z *denotes the topic responsible for generating that word, drawn from the *θ *distribution for that document, and *w *is the word itself, drawn from the topic distribution *ϕ *corresponding to *z*. *N_d _*stands for the number of words in the document. *D *stands for the size of document collection. Estimating *ϕ *and *θ *provides information about the topics in a collection and the weights of those topics in each document. A host of algorithms have been used to estimate these parameters, ranging from Mean field variational methods [11], Expectation propagation [17], Gibbs sampling [18], Collapsed variational inference [19] to Fast Collapsed Gibbs Sampling [20]. Under this unsupervised model, documents can be associated with multiple topics and we could automatically discover the topics covered by document collection.

#### Aspect distribution transformation

We construct *θ *matrix of Eq.(1) in light of LDA model discussed in above subsection, which is the matrix of passage-specific mixture weights for these *T *aspects discovered. *θ *provides the information about the aspects in the retrieved passage collection and the weights of those aspects in each retrieved passage. *θ_i _*denotes the aspects distribution for each passage *P_i_*. *a_ij _*stands for the weight of the aspect *A_j _*given the passage *P_i _*such that $\sum_{j=1}^{T}{a_{i}}_{j}$ equals one.

(1) $$\begin{array}{c} \theta =\;\;\;{\left(\theta_{1},\theta_{2},\cdots \;,\theta_{D}\right)}^{T}\\ \;\;\;\;=\left(\begin{array}{ccccc} a_{11} & \cdots & a_{1j} & \cdots & a_{1T}\\ \cdots & \cdots & \cdots & \cdots & \cdots \\ a1 & \cdots & a_{ij} & \cdots & {a_{i}}_{T}\\ \cdots & \cdots & \cdots & \cdots & \cdots \\ a_{D1} & \cdots & a_{Dj} & \cdots & a_{DT} \end{array}\right)\\ \;\;\;\;\;\left(1\leq i\leq D,1\leq j\leq T\right) \end{array}$$

We have observed the following two interesting phenomena from the column of matrix *θ*. First, for some specific aspects, majority passages of the retrieved collection get large weight values; however, for some other specific aspects, a few passages get large weight values. This suggests that for each aspect the distribution of the retrieved passages is different. Second, even the same weight value in different columns of *θ *matrix would have a different importance for different aspects. For instance, given two specific aspects *A_m _*and *A_n_*, there exist two weights *a_pm _*and *a_qn _*for passage *P_p _*and *P_q _*respectively, which satisfies *a_pm _*= *a_qn_*(1 *≤ p*, *q ≤ D*). If most of the passages have a smaller weight than *a_pm _*for aspect *A_m_*, and have a larger weight than *a_qn _*for aspect *A_n_*, then we can easily have a conclusion that it is more important of *a_pm _*for aspect *A_m _*than *a_qn _*for aspect *A_n_*. Therefore, we tend to make transformation of *θ *matrix to represent the importance of each passage in each aspect.

Given such a hypothesis that for each aspect, the importance of retrieved passages is a normal distribution, we can have *T *normal distributions, denoting by *N *= (*N*_1_, *N*_2_, ..., *N_T _*). Given an normal distribution *N_i _*(1 *≤ i ≤ T *), mean *μ_i _*and variance *σ_i _*are referred to Eqs.(2) and (3) respectively.

(2) $$\mu_{i}=\frac{\sum_{j=1}^{D}a_{ji}}{D}$$

(3) $$\sigma_{i}=\frac{\sum_{j=1}^{D}{\left(a_{ji}-\mu_{i}\right)}^{2}}{D}$$

where *a_ji _*stands for the weight of the aspect *A_i _*for passage *P_j _*, and *D *denotes the number of retrieved passages. In addition, we get a new matrix Θ shown as Eq.(4) to measure the passages. importance for each aspect.

(4) $$\begin{array}{c} \Theta =\;\;\;{\left(\Theta_{1},\Theta_{2}\cdots \;,\Theta_{T}\right)}^{T}\\ \;=\left(\begin{array}{ccccc} N_{1}\left(a_{11}\right) & \cdots & N_{1}\left(a_{j1}\right) & \cdots & N_{1}\left(a_{D1}\right)\\ \cdots & \cdots & \cdots & \cdots & \cdots \\ N_{i}\left(a_{1i}\right) & \cdots & N_{j}\left(a_{ji}\right) & \cdots & N_{i}\left(a_{Di}\right)\\ \cdots & \cdots & \cdots & \cdots & \cdots \\ N_{T}\left(a_{1T}\right) & \cdots & N_{T}\left(a_{jT}\right) & \cdots & N_{T}\left(a_{DT}\right) \end{array}\right)\\ \;\;\;\;\;\;\;\left(1\leq i\leq T,1\leq j\leq D\right) \end{array}$$

where *N_i_*(*a_ji_*) denotes the importance of passage *P_j _*for the aspect *A_i_*. Θ*_i _*denotes the importance distribution of passage collection for the aspect *A_i_*.

### Re-ranking with *N*-size slide window

Re-ranking problem is defined as this: Given a query *q *and an initial ranking *R *produced for this query only with respect to relevance, we build a new ranking *S *taking account of both relevance and diversity. In terms of ranking *R*, our aim is that given a cut o position *k *of *S*, top *k *passages of *S *could deliver as many non-redundant aspects as possible. In this section, we introduce two re-ranking algorithms based on a slide window to promote ranking diversity. We set the slide window with size *N*, and put top *N *passages from *R *as candidate passages into the slide window when re-ranking. As we commonly set *N *with a small number, we suppose that there is no distinctive difference between passages in a slide window with respect to their query-relevance.

First, we choose a passage from the slide window as the first passage in ranking *S*, which contains the largest aspect coverage as show in Eq.(5).

(5) $$MaxAspCoverg=\underset{q\in \left[1,N\right]}{arg max}\overset{T}{\underset{t=1}{\sum }}N_{t}\left(a_{tq}\right)$$

where *N_t_*(*a_tq_*) denotes the importance of passage *P_q _*for the aspect *A_t _*and $\sum_{t=1}^{T}N_{t}\left(a_{tq}\right)$ stands for the aspect coverage of passage *P_q_*. After adding this passage into ranking *S*, we remove it from ranking *R*. For the rest of passages in *R*, if the number of passages in *R *is not less than *N*, we will put the top *N *passages in *R *into the slide window, or else we will put all the passages in *R *into slide window. Then we choose a passage from the slide window, which contains the most distinctive aspects compared with the observed passages in ranking *S*, add it into *S*, and remove it from *R*. The working scheme of this ranking method based on *N *size slide window is described in Algorithm 1, named *rank_-_NWin*.

### Algorithm 1 rank_-_NWin Algorithm

1: **Input: **An initial passage ranking *R *produced for current user query only with respect to relevance, and the size *N *of the slide window

2: **Output: **A reranked passage list *S*

3: **Process:**

4: Given top *N *passages in R, we find a passage *pass*_1 _containing the most aspect coverage value using Eq.(5);

5: *R ← R\{pass*_1_*}*;

6: *S ← *Ø∪*{pass*_1_*}*;

7: **while ***R:length *≠ 0 **do**

8:   Choose top *N *passages in *R *as candidate passages and if the length of rank *R *is less than *N *, take all passages in *R *as candidate passages;

9:   **for **each passage *i *of candidate passages **do**

10:    *distance*_-_*R_i _*= 0;

11:    **for **each passage *j *in *S ***do**

12:       *distance*_-_*R_i _*= *distance*_-_*R_i _*+ *Distance*(*R_i_, S_j _*);

13:    **end for**

14:    *distance*_-_*R_i _*= *distance*_-_*R_i _/S.length*;

15: **end for**

16: Find the max *distance_R _*passage *pass_rest _*in candidate passages;

17: *R ← R\{pass_rest_}*;

18: *S ← S *∪*{pass_rest_}*;

19: **end while**

20: return *S*.

The advantage of the Algorithm 1 is that it considers aspect distinctions between candidate passages in the slide window and observed passages ranked in *S*. However, considering original query-relevance ranking *R*, it is not appropriate for Algorithm 1 to change in a wide range of *R*. Therefore, another algorithm named *rank_-_NWin_-_Group *is proposed to ensure that the new ranking *S *is just the original ranking *R *with slight adjustments. The key idea of this algorithm is described below. For the first passage in *S*, we still choose a passage containing the largest aspect coverage from the slide window, add it into *S *and remove it from *R*. Different from *rank_-_NWin *Algorithm, we first group rank *R *into several *N *size groups, and the size of last group may be less than *N*. We put each group into the slide window in turn, re-rank the passages in current group, and add them into *S *finally. The process of re-ranking in groups is similar to algorithm *rank_-_NWin*. Algorithm 2 describes the process of re-ranking by using *N*-size slide window to group ranking *R*.

### Algorithm 2 rank*_-_*NWin*_-_*Group Algorithm

1: **Input: **An initial passage ranking *R *produced for current user query only with respect to relevance, and the size *N *of the slide window

2: **Output: **A reranked passage list *S*

3: **Process:**

4: Given top *N *passages in R, we find a passage *pass*_1 _containing the most aspect coverage value using Eq.(5);

5: *R ← R\{pass*_1_*}*;

6: *S ← *Ø∪ *{pass*_1_*}*;

7: Group passages in *R *into [*R.length/N*] groups;

8: **for **each group *i ***do**

9:    **for **each passage *j *in group *i ***do**

10:       *distance_-_R_j _*= 0;

11:       **for **each passage *k *in *S ***do**

12:          *distance_-_R_j _*= *distance_-_R_j _*+ *Distance*(*R_j _. S_k_*);

13:       **end for**

14:       *distance_-_R_j _*= *distance_-_R_j _= S:length*;

15:    **end for**

16:    Rank passages in group *i *according to *distance_R _*in a descend order.

17:    *R ← R\{pass in group i}*;

18:    *S ← S *∪ *{pass in group i}*;

19: **end while**

20: return *S*.

*Distance*(*i, j*) in algorithms *rank_-_NWin *and *rank_-_NWin_Group *is the measurement of the aspect distinction between two passages. Given two passages, the more different the aspects are, the larger value of *Distance*(*i, j*) will be. In our work, we use two slightly different ways to evaluate it. The first one can be seen as the original Euclidean distance as shown in Eq.(6).

(6) $$Distance\left(i,j\right)=\sqrt{\overset{T}{\underset{t=1}{\sum }}{\left(N_{t}\left(a_{ti}\right)-N_{t}\left(a_{tj}\right)\right)}^{2}\;}\;\left(i\neq j\right)$$

Furthermore, we assume that the importance of each aspect is different, and *μ_t_*(1 *≤ t ≤ T *) as discussed in the last subsection denotes the mean distribution of the whole passage collection for the *A_t _*aspect. We thus regard *μ_t _*as the weight value and then get another equation for Euclidean distance as shown in Eq.(7).

(7) $$Distance{\left(i,j\right)}^{*}=\sqrt{\overset{T}{\underset{t=1}{\sum }}\mu_{t}{\left(N_{t}\left(a_{ti}\right)-N_{t}\left(a_{tj}\right)\right)}^{2}}\;\left(i\neq j\right)$$

## Results

### Dataset and evaluation metrics

In order to evaluate our proposed approach for promoting ranking diversity in biomedical information retrieval, we employ TREC 2007 Genomics track collection as the test data set. It is a full-text biomedical collection consisting of 162,259 documents from about 49 genomics-related journals indexed by MEDLINE [1,2]. These documents come from the Highwire Press (http://www.highwire.org) electronic distribution of journals and are in HTML format, which preserves the formatting, structure, table and figure legends, etc. There are 36 official topics for the track in 2007, which are in the form of questions asking for lists of specific entities that cover different portions of full answers. Here "topic" means "query" [1,2].

The followings are examples of queries from the 2007 Genomics Track:

• Query 200: What serum [PROTEINS] change expression in association with high disease activity in lupus?

• Query 221: Which [PATHWAYS] are mediated by CD44?

• Query 231: What [TUMOR TYPES] are found in zebrafish?

For TREC 2007 Genomics track, there are three levels of retrieval performance measured: passage retrieval, aspect retrieval, and document retrieval. Each of these provides insight into the overall performance for a user trying to answer the given questions. These three levels were measured by some variant of MAP. Passage MAP, Passage2 MAP, Aspect MAP and Document MAP, defined in [1] and [2], are four evaluation metrics corresponding to the three levels of retrieval performance. In this paper, we mainly focus on two evaluation metrics, Aspect MAP and Passage2 MAP, since our objective is to promote diversity in the ranked list of retrieved passages. Furthermore, aspect retrieval and passage retrieval are also the major tasks in TREC 2007 Genomics tracks.

Genomics collections only contain a fraction of millions of biomedical literatures indexed by MEDLINE, but as far as we know, they are the largest and the only biomedical text collections with both manual relevance assessments and diversity evaluation available for biomedical text retrieval research.

### Retrieval baselines

We employ two retrieval baseline runs, NLMinter [21] and UniNE2 [22]. NLMinter developed by U.S. National Library of Medicine achieved the best performance in TREC 2007 Genomics track in terms of Aspect MAP, Passage2 MAP and Document MAP. UniNE2 which is developed by University of Neuchatel Rue Emile-Argand combined different search strategies. This baseline run proposes a new approach to the generation of orthographic variants of search terms, and the generation of the I(n)B2 [23] with the article title included in each passage, or with both the article and orthographic variants. The performance of UniNE2 was above average among all results reported in TREC 2007 Genomics track.

### Re-ranking performance

We preprocessed the retrieved passages of two baseline runs. For instance, any delimiting character, including hyphen, was used to separate words, and we deleted any words that occurred only once in one passage or belonged to the standard "stop" list (http://www.link-assistant.com/seo-stop-words.html) used in Google retrieval engine.

Re-ranking results of the proposed methods on TREC 2007 Genomics collection are shown in Table 1 and Table 2. The values in the parentheses are the relative rates of improvement over the original results. It can be seen from the table that our approaches can make improvements over both baseline runs. For the efficiency reason, we re-ranked only the top 100 passages. Distinctive improvements over all baseline runs in terms of Aspect MAP can be observed.

**Table 1:** Re-ranking performance with NLMinter

MAP	Aspect	Passage2	Passage	Document
NLMinter	0.23068962	0.07335484	0.05971977	0.20962491

*rank_-_NWin*	0.2438342	0.07368625	0.05868155	0.20790886
	(+5.70%)	(+0.45%)	(-1.74%)	(-0.82%)
rank_-_NWin*	0.24426998	0.07372402	0.05849706	0.20744464
	(+5.89%)	(+0.50%)	(-2.05%)	(-1.04%)
*rank_-_NWin_-_Group*	0.24908569	0.07792334	0.06151813	0.20976964
	(+7.97%)	(+6.23%)	(+3.01%)	(+0.07%)
rank_-_NWin_-_Group*	**0.24910669**	0.07793161	0.06152586	0.20977025
	**(+7.98%)**	(+6.24%)	(+3.02%)	(+0.07%)

Table 1 shows the comparison of our proposed methods and NLMinter on TREC 2007 Genomics collection. *rank_-_NWin *and *rank_-_NWin_-_Group *represent two reranking approaches with *Distance*(*i, j*), Rank_-_NWin* and rank_-_NWin_-_Group* denote two reranking approaches with Distance* (*i, j*).

**Table 2:** Re-ranking performance with UniNE2

MAP	Aspect	Passage2	Passage	Document
UniNE2	0.09880169	0.01777397	0.05236709	0.13771527

*rank_-_NWin*	0.1052544	0.01946295	0.05459447	0.13969831
	(+6.53%)	(+9.50%)	(+4.25%)	(+1.44%)
rank_-_NWin*	0.1052544	0.01946007	0.05459788	0.13964510
	(+6.53%)	(+9.49%)	(+4.26%)	(+1.40%)
*rank_-_NWin_-_Group*	**0.10554020**	0.01902429	0.05490502	0.14035642
	**(+6.82%)**	(+7.03%)	(+4.85%)	(+1.92%)
rank_-_NWin_-_Group*	0.10549095	0.01902427	0.05490508	0.14035350
	(+6.77%)	(+7.03%)	(+4.85%)	(+1.92%)

Table 2 shows the comparison of our proposed methods and UniNE2 on TREC 2007 Genomics collection.

Re-ranking performance is effected by the parameters chosen from LDA model. Comparison with NLMinter baseline run, we only show the re-ranking results with parameters of *β *whose values are equal to 0.04 in algorithm 1 and 0.06 in algorithm 2, respectively. For UniNE2 baseline run, we show re-ranking results with parameters of *β *whose values are equal to 0.004 in algorithm 1 and 0.008 in algorithm 2, respectively. The choosing of the parameters in LDA will be discussed in the next subsection.

## Discussion

### Impact of parameter β

The statistical model LDA we have described is conditioned on three parameters, the Dirichlet hyper-parameters *α *and *β*, and the number of topics *T*. The choice of *α *and *β *can have important implications for the results produced by the model. In particular, the value of *β *affects the granularity of the model. Increasing *β *can be expected to decrease the number of topics used to describe retrieved passages. In other words, retrieved passages can be sensibly factorized into a set of topics at several different scales, and the particular scale of the topics assessed by the model will be set by *β*. A large value of *β *would lead the model to find a relatively small number of topics. Since we focus on biomedical domain, we tend to employ smaller values of *β*, which will result in more topics that address specific fields.

We should choose the value of *β *for each specific user query. In order to improve experimental efficiency, we choose *β *according to the retrieval passages instead. As preprocess give us two word sets of 10,222 words and 2,387 words for retrieved passages by NLMinter and UniNE2 baseline runs respectively, we give two different settings of *β*. For NLMinter, we set the values of *β *∈ [0.01, 0.08] in steps of 0.01. However, for baseline UniNE2, we set the values of *β *∈ [0.002, 0.009] in steps of 0.001. These values of *β *are relatively small and can be expected to give rise to a fine-grained decomposition of the collection into topics that address specific research fields.

### Impact of parameters α and T

Given values of *β*, the problem of choosing appropriate values for *α *and *T *thus is a problem of model selection. We let *αT *= *constant *to keep constant the sum of the Dirichlet hyper-parameters, which can be interpreted as the number of virtual samples contributing to the smoothing of *θ *[18]. Moreover, because our strategy in this article is to fix *αT *= *constant*(Here, we set *constant *= 10 in order to make *α *not larger than 1 [18].) and *β*, and explore the consequences of varying *T *, for each fixed *β *value we set the values of *T *from 10 to 100 in steps of 10 consecutively. To evaluate the consequences of changing the number of topics *T *, we used the Gibbs sampling algorithm [18] to obtain samples from the posterior distribution over *z *at several choices of *T*.

Next, we need to choose an appropriate value of *T *for each specific query. In our case, the data are the words in the retrieved passages, *w*, and the model is specified by the number of topics, *T *, thus we wish to compute the likelihood *p*(*w|T *). However, this requires to sum over all possible assignments of words to topics *z*. We can approximate *p*(*w|T *) by the harmonic mean of a set of values of *p*(*w|z, T *) when *z *is sampled from the posterior *p*(*z|w, T *) [18]. In all cases, *p*(*w|T *) increases at the beginning, and decreases after reaching a peak.

**Figure 2:**
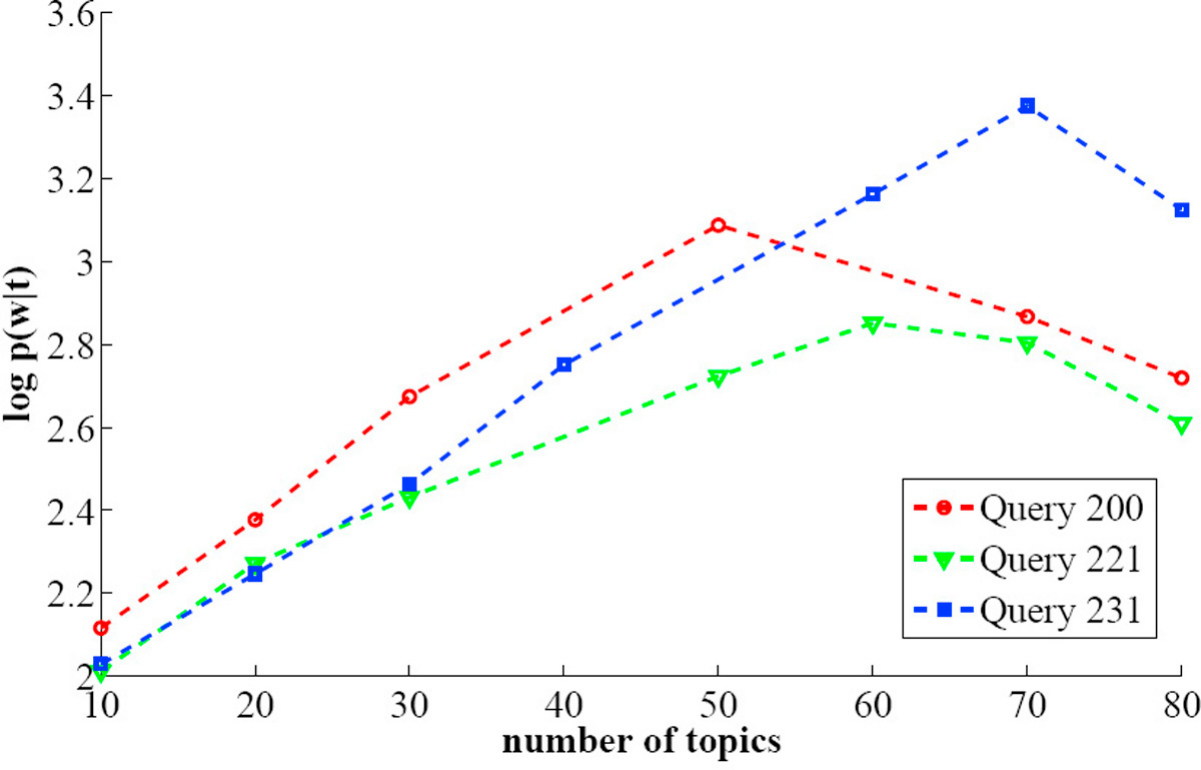
**Model selection results**. Figure 2 shows the log-likelihood of the data for different settings of the number of topics *T *for query 200, 221 and 231 with *β *being equal to 0.06.

Figure 2 shows the log-likelihood of the data for different settings of the number of topics *T *for query 200, 221 and 231 in our data collection with *β *being equal to 0.06. For example, for query 200, the results suggest that the data are best accounted for by a model incorporating 50 topics. *p*(*w|T *) initially increases as a function of *T *, reaches a peak at *T *= 50, and then decreases thereafter.

As mentioned above, the value of *T *depends on the choices of *α *and *β*, which will also be affected by specific decisions made in forming the dataset such as the use of stop-word list, etc. The distribution over topics illustrates how this statistical model can capture similarity in the semantic content of documents.

### Comparison of the two reranking strategies

**Figure 3:**
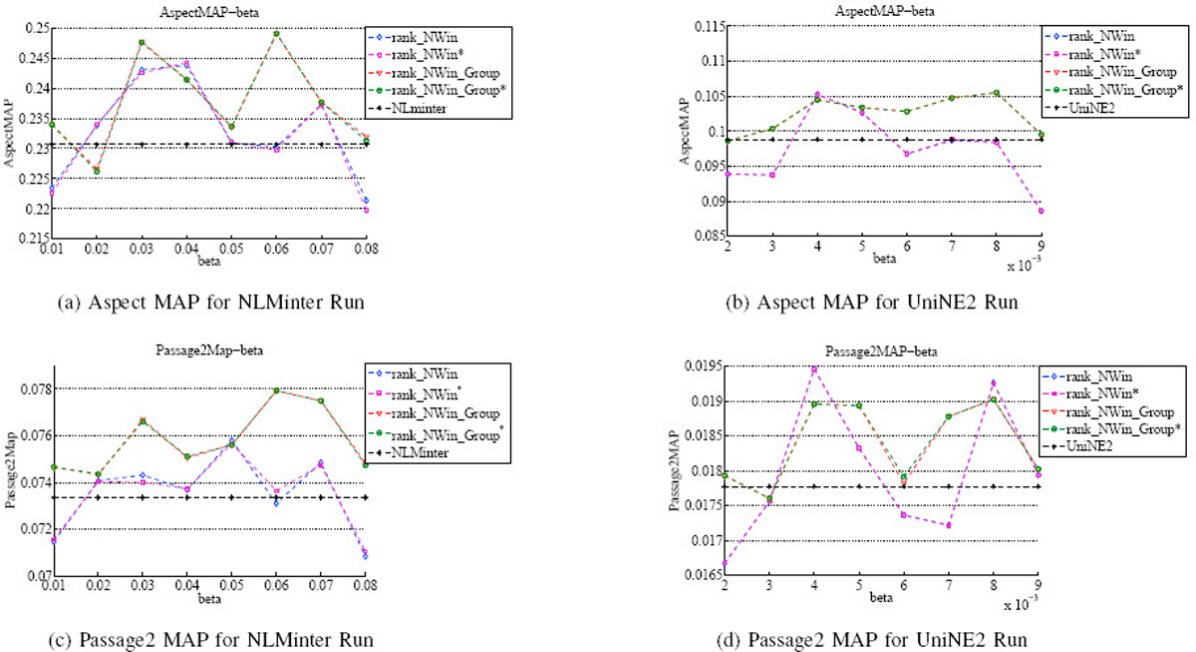
**Impact of ***β ***parameter**. Figure 3 shows NLMinter and UniNE2 system performance with varying *β*.

The parameter *β *indicates the scale of topics for the retrieved passages. Given different *β*, retrieved passages can be factorized into a series of topics at different scales. We propose two re-ranking algorithms and two distance metrics, and therefore have four re-ranking algorithms, whose re-raking performance can be also shown in Figure 3. As the aspect level retrieval and the passage level retrieval were two major tasks in the TREC 2007 Genomics tracks, system performances at these two levels with different *β *are also shown in Figure 3.

Figures 3(a) and 3(b) respectively show NLMinter and UniNE2 system performances at aspect level with different *β*. It can be seen from Figure 3(a) that when *β*'s value is between 0.03 and 0.07, performance improvements on aspect level can be achieved for all re-ranking strategies. For ranking strategies *rank_-_NWin *and rank_-_NWin* , the Aspect MAP increases with the increase of *β*, reaches at a peak for *β *= 0.04, then decreases, and reaches at a local peak when *β *= 0.07, and finally it plummets. For *rank_-_NWin_Group *and rank_-_NWin_Group*, the Aspect MAP increases from *β *= 0.02, reaches at a local peak when *β *= 0.03, then drops down and jumps to a peak for *β *= 0.06, and thereafter falls down. Figure 3(b) shows that retrieval results at aspect level are better than the baseline runs with all *β*s for *rank_-_NWin_Group *and rank_-_NWin Group*. Aspect Map increases as *β *increases, reaches a local peak when *β *= 0.004, and then decreases slightly, after that grows when *β *= 0.006, reaches the maximum, and then drops down quickly. For *rank_-_NWin *and rank_-_NWin* , the performance improvements on aspect level are achieved when *β *= 0.004 and 0.005.

NLMinter and UniNE2 system performances at passage level with different *β *are shown in Figures 3(c) and 3(d). Comparing Figures 3(a) and 3(b) with Figures 3(c) and 3(d) respectively, we could observe that the trends of performances on aspect level and passage level are generally in agreement with *rank_-_NWin_-_Group *and rank_-_NWin_Group*. The observation illustrates that there are a clear correlation between Aspect MAP and Passage MAP. However, for *rank_-_NWin *and rank_-_NWin*, the trends of performances on aspect level is different from passage level. This could be caused by the reason that *rank_-_NWin *and rank_-_NWin* algorithms change original passage ranking within a large ranges. Furthermore, we demonstrate that two different distance metrics, with or without weight, do not influence re-ranking performance significantly.

The comparison results shown in Figure 3 indicate that both of the two proposed re-ranking methods are effective in promoting diversity for biomedical information retrieval, and *rank_-_NWin_-_Group *outperforms *rank_-_NWin *in most cases.

## Conclusions

In this paper, we propose an approach which employs LDA, a topic generative model, to promoting ranking diversity for biomedical information retrieval. Our contribution is three-fold. First, to the best of our knowledge, this is the first study of adopting topic model to biomedical IR. Different from other approaches considering aspects on word level, our approach assumes that aspects should be identified by the topics of retrieved documents. We employ LDA model to discover topic distribution of retrieval passages and word distribution of each topic dimension. Second, since retrieved passages' distribution for each aspect is different, even the same weight value in different aspects would be of different importance, we made transformations with topic distribution. Third, two re-ranking algorithms based on "*N*-size slide window" are proposed, which take both passage novelty and relevance into account. Experiments conducted on TREC 2007 Genomics track collection demonstrate the e effectiveness of our approach. The evaluation results show that our approach can achieve 8% improvement over the highest Aspect MAP reported in TREC 2007 Genomics track.

In future research, we intend to extend this work by exploring both more complex models and more sophisticated algorithms. We have shown the e effectiveness of our approach in biomedical information retrieval area, and this approach can be adopted to a variety of other domains. For example, we could apply our approach to other test collections, such as ClueWeb09 collection, to investigate whether the approach is still effective for improving ranking diversity in the Web search. Furthermore, ranking diversity plays an important role in a range of tasks or applications, such as information retrieval, social network analysis and recommendation system, etc. We thus plan to further improve our approach to solve the diversification in the above mentioned fields.

## Competing interests

The authors declare that they have no competing interests.

## Authors' contributions

YC proposed reranking strategies, carried on the experiments and drafted the manuscript. XY and JXH contributed in the study design and experiments. ZL, XH and JXH supervised the study and revised the manuscript.
